# The ageing meniscus: Pathophysiology, epidemiology and management

**DOI:** 10.1002/jeo2.70799

**Published:** 2026-06-15

**Authors:** Alberto Grassi, Claudio Rossi, Stefano Zaffagnini, Peter Verdonk

**Affiliations:** ^1^ Clinica Ortopedica e Traumatologica II IRCCS Istituto Ortopedico Rizzoli Bologna Italy; ^2^ Dipartimento di Scienze Biomediche e Neuromotorie (DIBINEM) Università di Bologna Bologna Italy; ^3^ OrthoCA Orthopaedic Center Antwerp Belgium; ^4^ Department of Orthopaedic Surgery Antwerp University Hospital Edegem Belgium

**Keywords:** ageing, conservative management, degenerative tear, meniscus, orthobiologics, osteoarthritis

## Abstract

The meniscus is a fibrocartilaginous structure crucial for load distribution, shock absorption and knee stability. With advancing age, its biomechanical and biochemical integrity declines through progressive cellular senescence, accumulation of advanced glycation end‐products, reduced vascularity and altered collagen cross‐linking. These changes predispose to structural damage most often seen as *horizontal cleavage lesions*—the hallmark of meniscal ageing—best understood as ‘wrinkles’ of the meniscus rather than true pathology. Such lesions are often asymptomatic and incidental on MRI. However, in some individuals they may cause pain or mechanical symptoms requiring targeted, conservative management. This narrative review integrates biological, epidemiological and clinical data to provide an evidence‐based framework for managing the ageing meniscus. We discuss the natural history of degenerative lesions and detail practical strategies for orthopaedic clinicians, including exercise therapy, metabolic optimization, nutritional supplementation, biomechanical offloading and injection‐based interventions. Surgical treatment should remain a second‐line option, reserved for patients with persistent symptoms or biomechanically significant lesions.

**Abbreviations**
5STS: 5‐Repetition Sit‐to‐Stand Test
30s‐STS: 30‐Second Sit‐to‐Stand Test
AGEs: advanced glycation end‐products
BMI: body mass index
CRP: C‐reactive protein
DFO: distal femoral osteotomy
ESWT: extracorporeal shockwave therapy
HA: hyaluronic acid
HDL: high‐density lipoprotein
HTO: high tibial osteotomy
IA: intra‐articular
KL: Kellgren–Lawrence
LDL: low‐density lipoprotein
LWI: laterally wedged insoles
MRI: magnetic resonance imaging
OA: osteoarthritis
PCS: Pain Catastrophizing Scale
PROMs: patient‐reported outcome measures
PRP: platelet‐rich plasma
TKA: total knee arthroplasty
TSK: Tampa Scale of Kinesiophobia
UKA: unicompartmental knee arthroplasty

## INTRODUCTION

Meniscal degeneration represents one of the earliest and most frequent manifestations of knee ageing. Over recent decades, robust evidence has demonstrated that meniscectomy accelerates the onset and progression of tibiofemoral osteoarthritis (OA) [55]. This association has sometimes led to the perception that postoperative degenerative changes are always a direct consequence of surgical intervention, implicitly attributing to a surgical act. However, emerging evidence indicates that certain meniscal lesions themselves may act as biological triggers of joint degeneration, independently of whether they are surgically resected or left untreated [15, 16, 17, 23, 47]. A longitudinal study of 347 knees from the Multicenter Osteoarthritis Study (MOST) demonstrated that meniscal tears and extrusion were strong predictors of fast cartilage loss within 30 months of follow‐up [48]; this damage was more evident in patients with high body mass index (BMI) and with joint effusion [46].

Therefore, it could be argued that in such specific cases, the degenerative cascade may be initiated by intrinsic meniscal pathology and matrix deterioration rather than by mechanical factors or surgical management. This suggests that in some patients, the process is inherently irreversible and reflects the natural course of knee and *meniscal ageing*.

This concept is important because clinicians should not limit their focus to the meniscal tear as an isolated structural defect but rather aim to understand the broader biological context of meniscal degeneration. Thus, being conscious of the natural history of these lesions, along with the ability to identify which lesion—and above all, which patient—will benefit from conservative versus surgical management, is essential for optimizing outcomes and avoiding overtreatment.

This awareness has led to a paradigm shift: degenerative meniscal lesions could be recognized as an age‐related phenomenon rather than purely mechanical injuries. In a *BJSM* editorial, Risberg [43] described degenerative meniscus tears as ‘wrinkles’, highlighting the role of the ageing process in this scenario.

Among all degenerative patterns, horizontal cleavage lesions are the most characteristic of ageing [2].

They arise through intrinsic matrix deterioration rather than trauma, often extending parallel to the tibial plateau and separating the superior and inferior lamellae. These horizontal lesions are frequently incidental on MRI and may remain asymptomatic for years [7, 16]. Like wrinkles on the skin, they reflect biological ageing rather than disease per se. Nevertheless, a subset of patients experiences pain, swelling or mechanical symptoms, particularly in the presence of meniscal extrusion or concomitant OA [5, 46].

The growing understanding of meniscal ageing and the recognition of degenerative lesions as part of this continuum have also prompted scientific societies to take a stance to propose clinical practice guidelines. As an example, the high prevalence of these horizontal degenerative tears and the controversies regarding their optimal management led the European Society of Sports Traumatology, Knee Surgery and Arthroscopy (ESSKA) to develop comprehensive, evidence‐based recommendations that classify and contextualize horizontal degenerative meniscal lesions (Table 1) [2]. Importantly, these guidelines emphasize that first‐line treatment should be conservative, reserving surgery for selected patients with persistent, functionally limiting symptoms. This position underscores the importance of prudence and clinical judgement when considering arthroscopic intervention for lesions that often represent a natural manifestation of the ageing process rather than a purely mechanical defect.

**Table 1:** Extract from the ESSKA consensus on degenerative meniscal lesions.

Question	Answer	Evidence
What is a degenerative meniscus lesion?	A degenerative meniscus lesion is a slowly developing lesion, typically involving a horizontal cleavage of the meniscus in a middle‐aged or older person. Such meniscus lesions are frequent in the general population and are often incidental findings on knee MRI. The pathogenesis is not fully understood. There is often no clear history of an acute knee injury.	(Grade B)
Do degenerative meniscus lesions cause knee symptoms?	There is very limited evidence that pain in the degenerative knee is directly attributable to a degenerative meniscus lesion even if the lesion is considered to be unstable. Great caution must be taken before arriving at the conclusion that the degenerative meniscus lesion is the direct cause of the patients' knee symptoms.	(Grade B)
Does an unstable degenerative meniscus lesion cause knee symptoms?	While there is limited support in the literature that degenerative meniscus lesions considered to be unstable, for example, flap tears, are truly causing knee symptoms, it is still plausible that, in some patients, torn meniscus parts from the degenerative lesion (by its displacement) may cause knee joint symptoms.	(Grade C)
What are the consequences of a degenerative meniscus lesion in the knee?	Loss of meniscus function may negatively affect the knee in the long term. Therefore, in many people, the degenerative meniscus lesion (which may impair the force transmission and load distribution capabilities of the meniscus) is a feature indicative of a knee joint with (or at increased risk of) developing OA.	(Grade B)
Are degenerative meniscus lesions a cause or consequence of knee OA?	The answer to this question is still unclear. However, one causal pathway does not necessarily exclude the other; that is, one phenotype of knee OA may start with meniscus degradation and degenerative lesions leading to loss of meniscus function and OA development. In turn, OA and its general degradation of the knee joint, involving multiple structures, may also cause degenerative meniscus lesions and extrusion that further accelerate structural progression of the disease.	(Grade B)
When should arthroscopic partial meniscectomy be proposed?	1. Surgery should not be proposed as a first line of treatment of Degenerative Meniscal Lesions.	(Grade A)
	2. Arthroscopic Partial Meniscectomy may be proposed after 3 months and persistent pain and/or mechanical symptoms related to a DML with normal X‐rays but an abnormal MRI (Grade III meniscus lesion). The patient has to be informed about chances of successful outcomes and risks of either method.	(Grade B)
	3. Surgery can be proposed earlier for patients presenting considerable mechanical symptoms. The patient has to be informed of chances and risks of either method.	(Grade D)
	4. No arthroscopic surgery should be proposed for degenerative meniscal lesions with advanced OA on weight‐bearing radiographs.	(Grade A)
	An exception should be discussed for young patients with considerable symptoms.	

*Note*: Extract from the ESSKA Consensus on Degenerative Meniscal Lesions: key statements regarding definition, clinical relevance and surgical indications with corresponding levels of evidence.

Abbreviations: ESSKA, European Society of Sports Traumatology, Knee Surgery and Arthroscopy; MRI, magnetic resonance imaging; OA, osteoarthritis.

Thus, understanding the biological basis of meniscal ageing allows clinicians to differentiate benign, stable changes from lesions that contribute to articular degeneration.

## PATHOPHYSIOLOGY OF MENISCAL AGEING

**Figure 1:**
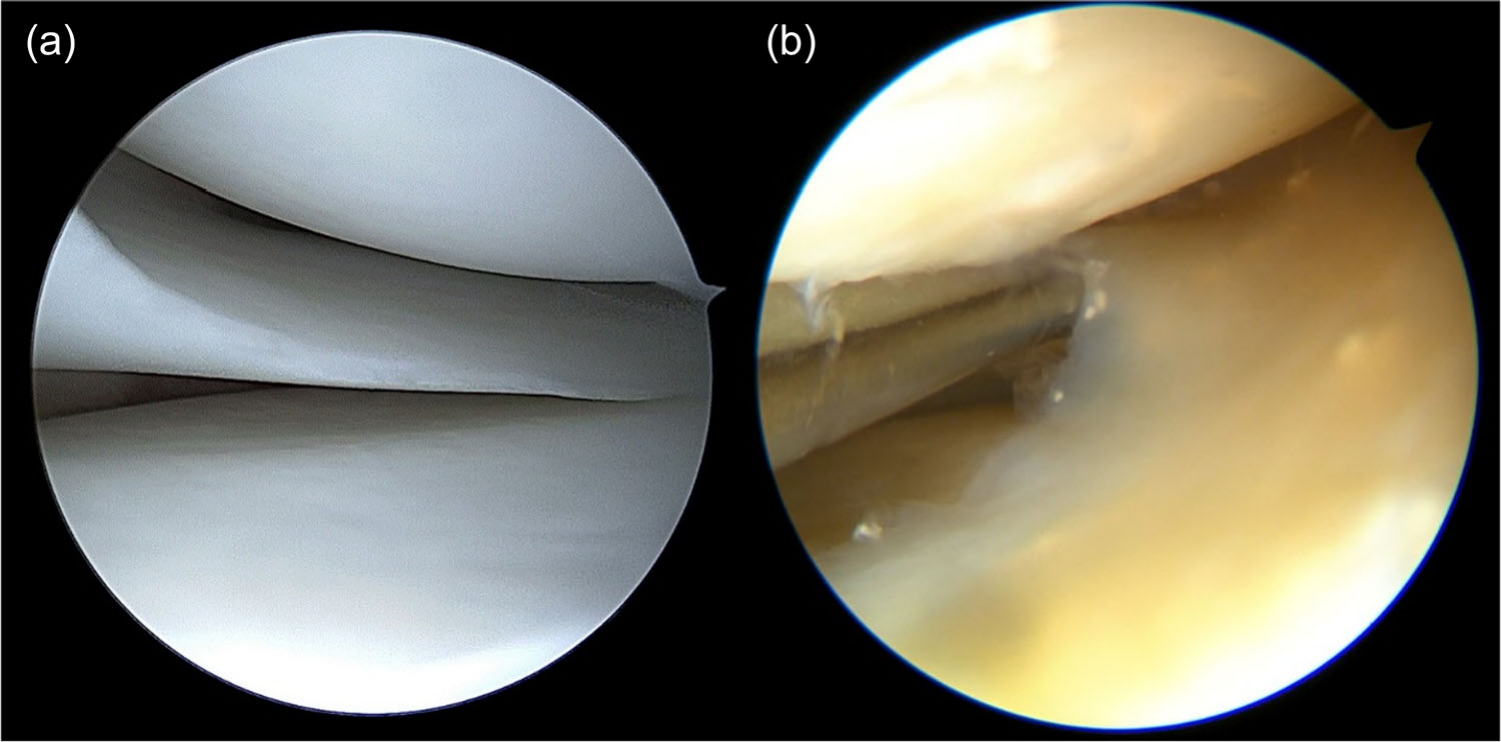
Arthroscopic appearance of young and healthy meniscal cartilage (a) compared to yellowish and fibrillated degenerated meniscal cartilage (b).

With advancing age, the meniscus undergoes profound morphological and biochemical remodelling that progressively impairs its mechanical integrity and reparative potential. Macroscopically, the healthy, translucent and glistening fibrocartilaginous tissue seen in youth becomes opaque and yellowish‐brown due to non‐enzymatic glycation of collagen fibres, known as the Maillard reaction [33, 53]. This accumulation of advanced glycation end‐products (AGEs) stiffens the matrix and diminishes its elasticity, producing a brittle tissue more vulnerable to microtrauma. The surface often appears roughened or finely fibrillated, reflecting gradual loss of fibronectin and other adhesive glycoproteins within the superficial extracellular matrix (Figure 1). Despite these surface changes, the overall meniscal shape is typically preserved until more advanced stages of degeneration [35, 40].

Microscopically, aging is characterized by reduced cellularity, increased fibrillar condensation and accumulation of mucoid material within the matrix. The extracellular matrix exhibits increased Safranin‐O staining, indicating a phenotypic shift from fibroblastic to chondrocytic cells, particularly within the inner avascular zone. Collagen fibres become thicker and more irregular, with enhanced non‐enzymatic cross‐linking through pentosidine bridges, further decreasing the tissue's capacity to deform under load. These biochemical and structural alterations collectively reduce the meniscus's ability to convert compressive forces into circumferential hoop stresses, predisposing it to intrinsic delamination [40].

**Figure 2:**
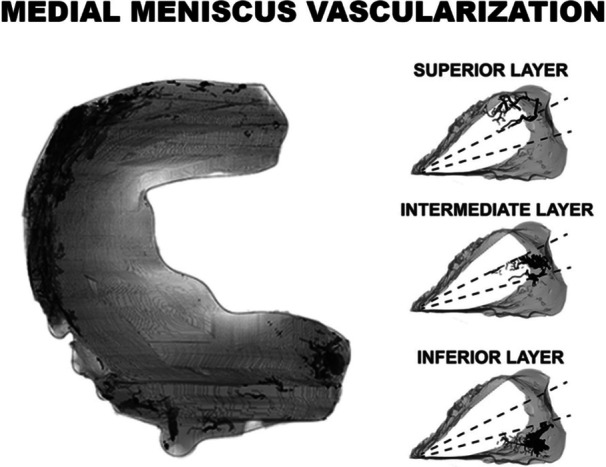
Axial and radial view of medial meniscus vascularization visualized by micro‐computed tomography (micro‐CT scan). With ageing, vascular supply becomes progressively restricted to the peripheral red–red zone, while the central white–white zone remains avascular and dependent on synovial diffusion.

Another critical element in the pathogenesis of meniscal aging is the progressive reduction in vascularization. The foetal meniscus is fully vascularized, but by adolescence only the outer 10%–30%—the so‐called red–red zone—receives direct blood supply from the perimeniscal capillary plexus. With age, this perfused region diminishes to approximately the peripheral quarter of the meniscus, leaving the central and inner white–white zones dependent solely on diffusion from synovial fluid (Figure 2) [37]. The declining vascularity limits nutrient delivery, waste clearance and cellular repair capacity, particularly after microinjury. Consequently, intrinsic healing potential becomes markedly restricted, and even small degenerative clefts may fail to resolve, predisposing to lesion propagation. Moreover, the intermediate layer of the medial meniscus has been demonstrated to be the poorer area of vascularization [38]. This avascular microenvironment also underlies the poor healing outcomes observed after meniscal repair in elderly patients.

The classic manifestation of these changes is the horizontal cleavage lesions, which represent the main manifestation of meniscal aging. The genesis of these lesions does not arise from acute trauma but could be due to chronic matrix fatigue along the lamellar planes weakened by collagen disorganization and proteoglycan imbalance. Within the substance of the meniscus, mucoid degeneration and focal accumulation of proteoglycans create cystic zones that separate the superior and inferior lamellae, forming horizontal clefts parallel to the tibial plateau [29]. Clinically, these lesions often remain asymptomatic and may be incidental findings on MRI. However, when propagation reaches the peripheral rim or the transition zone from the meniscal body to the ligamentous tibial attachment can alter load distribution and become symptomatic, manifesting as pain, effusion or mechanical discomfort.

**Figure 3:**
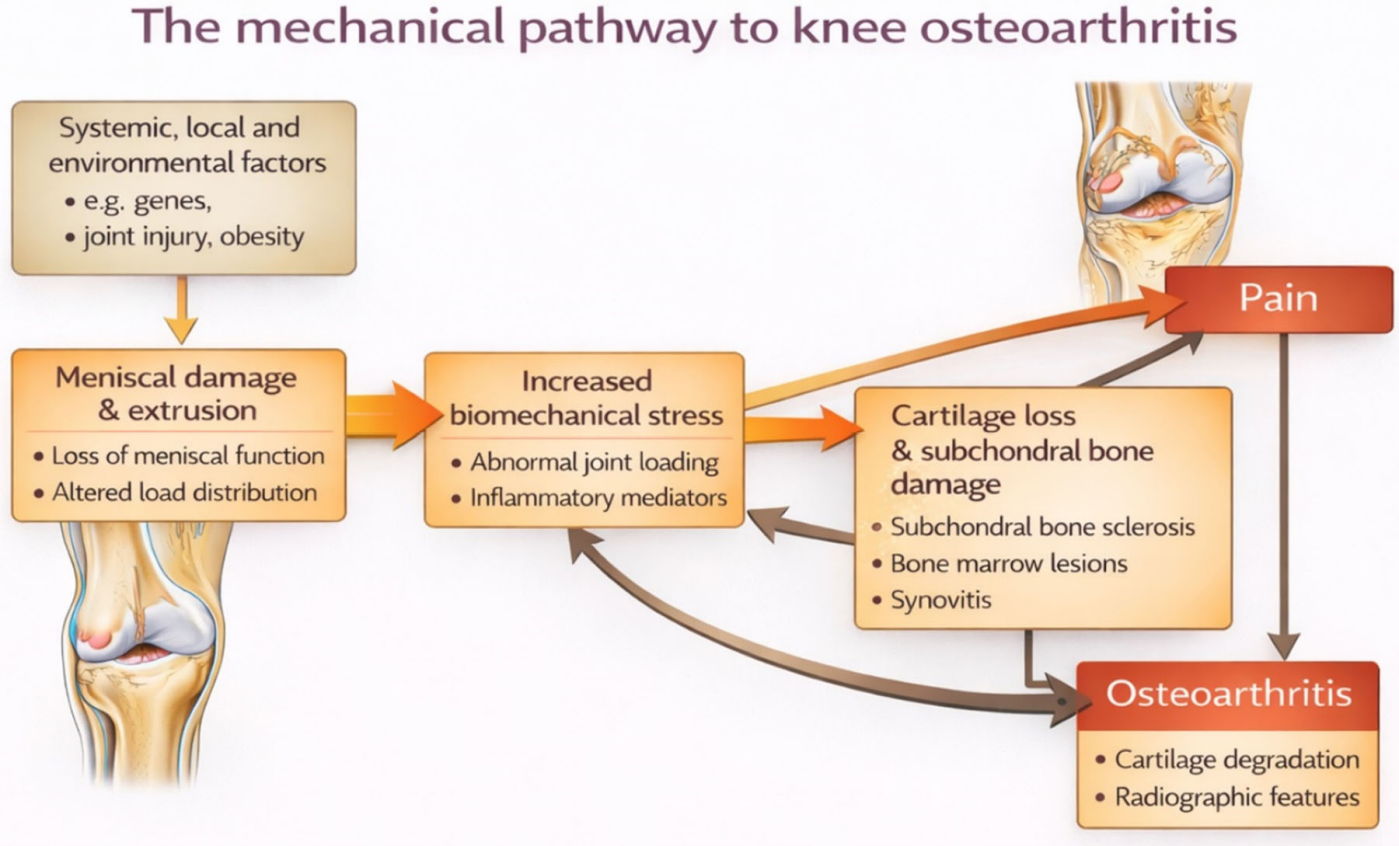
Proposed mechanical pathway linking age‐related meniscal degeneration to the development of knee osteoarthritis. Systemic and local factors, including aging, genetic predisposition, prior joint injury and metabolic influences, contribute to meniscal damage and extrusion. Loss of meniscal function alters load distribution across the tibiofemoral joint, leading to increased biomechanical stress, abnormal joint loading and inflammation. This cascade promotes cartilage loss, subchondral bone remodelling, synovitis and ultimately the structural and symptomatic progression of osteoarthritis.

Meniscus ageing thus represents a continuum between molecular senescence and structural degeneration. Reduced vascularity, glycation‐induced stiffening and matrix disorganization culminate in characteristic horizontal cleavage lesions and root failures. These changes may initiate a cascade of biomechanical and metabolic alterations within the whole knee joint (Figure 3), where the loss of meniscal integrity leads to increased focal stress on articular cartilage, subchondral bone remodelling and the development of bone marrow lesions, thereby perpetuating the osteoarthritic process [5, 15, 16, 49].

## EPIDEMIOLOGY AND NATURAL HISTORY

**Figure 4:**
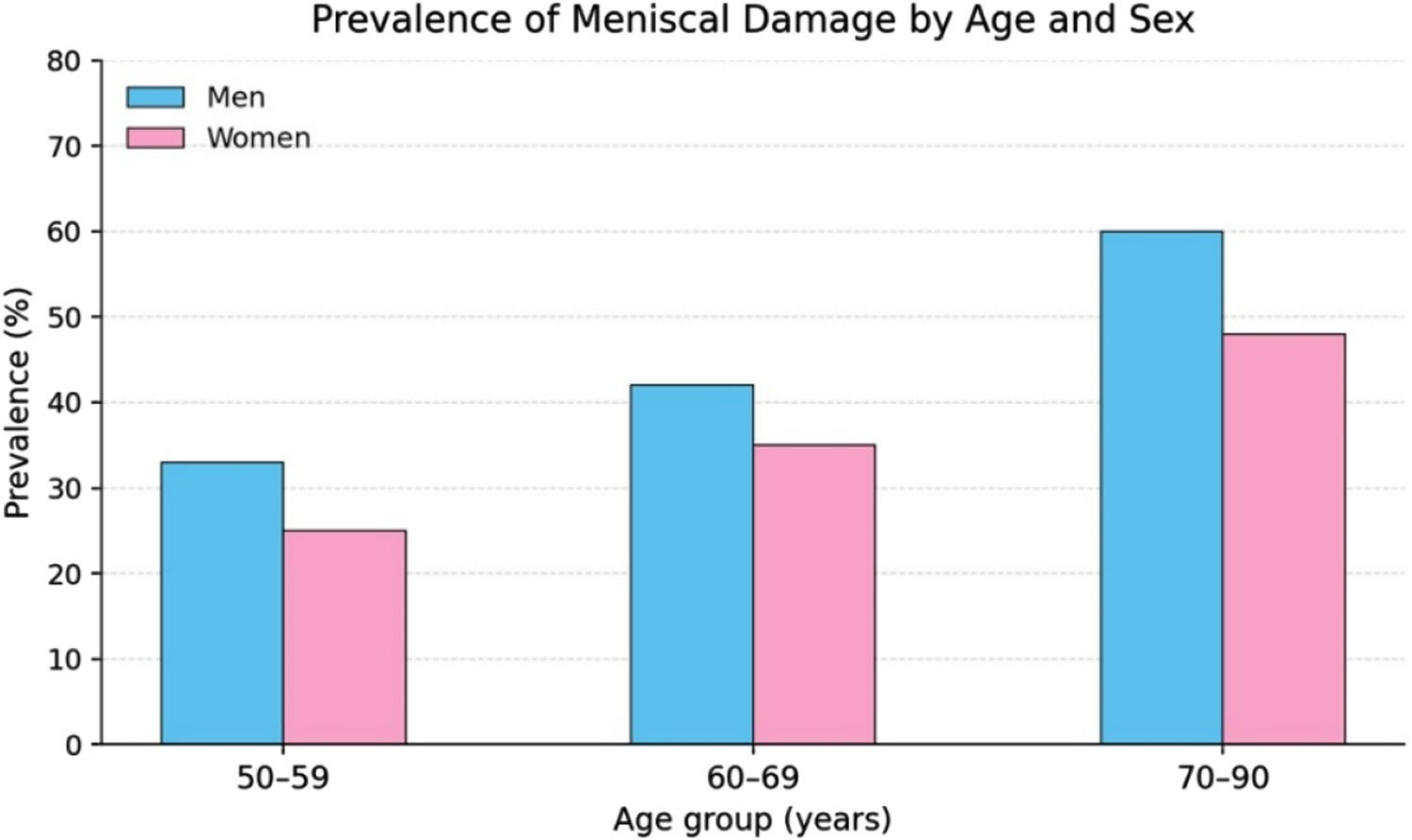
Bar chart showing age‐dependent increase in MRI‐detected meniscal tears in asymptomatic individuals. MRI, magnetic resonance imaging.

Data from autopsy analyses and magnetic resonance imaging (MRI) studies, including evaluations of contralateral asymptomatic knees, have demonstrated that meniscal lesions frequently occur in individuals without clinical symptoms [4, 13, 36]. Evidence from community‐based cohorts has further highlighted the widespread nature of this process. In the Framingham study, which examined adults aged 50–90 years without prior knee complaints, the prevalence of meniscal tears increased markedly with age, ranging from approximately 19% in women aged 50–59 years to more than 50% in men aged 70–90 years [16]. Moreover, around 10% of participants exhibited partial or complete loss of normal meniscal tissue, which is typically associated with radiographic OA rather than isolated meniscal injury (Figure 4). Similar observations have been reported in other population‐based MRI studies, confirming that meniscal abnormalities are common even among asymptomatic individuals and are, therefore, an almost inevitable feature of the ageing knee [7, 24].

Longitudinal studies also indicate that most degenerative lesions remain stable over time. Posadzy et al. [41] observed that 70% of horizontal lesions did not enlarge or provoke OA progression at 4 years. Risk factors for degenerative meniscal lesions extend beyond local trauma and were identified in systemic and biomechanical factors such as generalized OA (manifested by multiple bony enlargements of the finger joints), varus limb alignment and occupational exposure to repetitive kneeling or squatting as predictors of meniscal damage [16, 49].

Although many degenerative meniscal changes remain clinically silent, some may still contribute to the early stages of OA development. In fact, other longitudinal studies reported that the presence of a degenerative meniscal lesion on MRI was associated with a nearly six‐fold increased risk of developing radiographic OA over a 30‐month period [18, 46, 48], suggesting that meniscal deterioration may serve as both a marker and a mediator of early joint degeneration, even in the absence of pain. Specifically, tears involving the meniscal root—especially the medial posterior root—may play a relevant role in OA development since root detachment disrupts circumferential hoop stresses, causing extrusion and rapid cartilage wear, functionally equivalent to total loss of meniscus function [20, 23].

Metabolic comorbidities may influence degenerative processes as well: elevated serum cholesterol and triglycerides were reported to predict incident bone marrow lesions [9], while Vitamin D insufficiency has been correlated with greater cartilage and meniscal MRI abnormalities [27]. These findings reinforce the systemic dimension of joint ageing, linking cardiovascular and musculoskeletal health.

Finally, in individuals with symptomatic knee OA, the prevalence of meniscal pathology is even higher, with MRI evidence of degenerative tears in 70%–90% of cases [7, 23, 24]. However, the relationship between these imaging findings and clinical symptoms in the context of OA is complex and not fully understood.

## ASSESSMENT OF PATIENTS WITH MENISCAL AGEING

A comprehensive clinical assessment is fundamental for differentiating symptomatic degenerative meniscal lesions from incidental, age‐related findings. The evaluation should integrate medical history, functional performance, metabolic status and imaging, allowing the clinician to define both the biological context and the mechanical environment of the knee (Table 2).

**Table 2:** Comprehensive assessment of patients with meniscal ageing.

Domain	Assessment components	Prameters	Clinical implications
Clinical History and Examination	Symptom onset, pain characteristics, mechanical symptoms, comorbidities, medication review	Gradual onset, intermittent or activity‐related joint‐line pain; true locking uncommon; frequent association with obesity, metabolic syndrome, diabetes, hyperlipidemia, Vitamin D deficiency; use of corticosteroids or statins	Differentiates degenerative meniscal lesions from acute traumatic tears; identifies systemic contributors to joint ageing
Metabolic and Laboratory Assessment	Bone and cartilage metabolic status; systemic inflammation	25‐hydroxyvitamin D ( < 30 ng/mL insufficiency; <20 ng/mL deficiency); lipid profile (total cholesterol, LDL, HDL, triglycerides); HbA1c; CRP	Contextualizes meniscal degeneration within musculoskeletal ageing; supports targeted metabolic and nutritional interventions
Imaging—Radiography	Limb alignment and structural osteoarthritis	Standing long‐leg weight‐bearing radiographs; varus or valgus malalignment (>4° from neutral); Kellgren–Lawrence grading; joint space narrowing	Assesses mechanical loading environment; informs offloading strategies and surgical decision‐making
Imaging—Magnetic Resonance Imaging	Meniscal morphology, cartilage integrity, subchondral bone, synovitis	Horizontal cleavage or complex degenerative tears; early cartilage thinning; bone marrow lesions; meniscal extrusion ≥3 mm	Characterizes severity and pattern of meniscal degeneration; identifies osteoarthritis phenotypes and prognostic trajectories
Functional Performance Assessment	Lower‐limb strength, endurance, neuromuscular control	30‐Second Sit‐to‐Stand (30s‐STS); 5‐Repetition Sit‐to‐Stand (5STS); comparison with age‐ and sex‐matched normative values	Quantifies functional reserve; predicts outcomes following conservative or surgical management
Psychological Assessment	Pain‐related cognitions and fear‐avoidance behaviour	Pain Catastrophizing Scale (PCS); Tampa Scale of Kinesiophobia (TSK ≥ 37)	Identifies psychosocial factors contributing to pain and disability beyond structural findings; supports biopsychosocial management

*Note*: Comprehensive assessment of patients with degenerative meniscal lesions integrates clinical history and examination, metabolic status and laboratory tests, imaging evaluation, functional performance assessment and psychological assessment, in order to contextualize meniscal findings and promote phenotypic stratification and individualized management.

Abbreviations: CRP, C‐reactive protein; HbA1c, haemoglobin A1c; HDL, high‐density lipoprotein; LDL, low‐density lipoprotein.

### Clinical history and assessment

The initial step involves a detailed history focused on symptom onset, pain pattern and mechanical features. Degenerative lesions typically present with gradual onset of medial joint‐line discomfort, often described as intermittent or activity‐related rather than acute. Locking or true mechanical blockage is uncommon and, when present, suggests a flap or displaced fragment rather than a simple horizontal cleavage tear.

A comprehensive medical review is essential, as systemic comorbidities frequently coexist with degenerative meniscal pathology. Particular attention should be given to high BMI (>25) or metabolic syndrome, hyperlipidemia, obesity, diabetes mellitus and Vitamin D deficiency, all of which contribute to joint ageing and structural vulnerability [9, 27]. Medication history should be examined for agents affecting bone and cartilage metabolism, such as corticosteroids or statins.

### Blood tests

Baseline blood tests are recommended to identify metabolic contributors to meniscal and osteoarticular degeneration. Vitamin D (25‐hydroxyvitamin D) values below 30 ng/mL are considered insufficient and warrant supplementation, with severe deficiency (<20 ng/mL) that may correlate with increased pain and worse MRI findings [27]. Total cholesterol, low‐density lipoprotein (LDL), high‐density lipoprotein (HDL) and triglycerides should be assessed as well, since elevated levels have been reported to predict subchondral bone marrow lesions and may accelerate joint degeneration [9]. Finally, glycemic status (HbA1c) and C‐reactive protein (CRP) can be assessed when systemic inflammation or metabolic syndrome is suspected.

These metabolic data help to frame the meniscal lesion within the broader context of musculoskeletal ageing and guide interventions such as vitamin D repletion, dietary counselling or lipid‐lowering therapy.

Imaging remains pivotal for characterizing the morphology of meniscal lesions and assessing alignment and concomitant degenerative changes.

### Imaging

**Figure 5:**
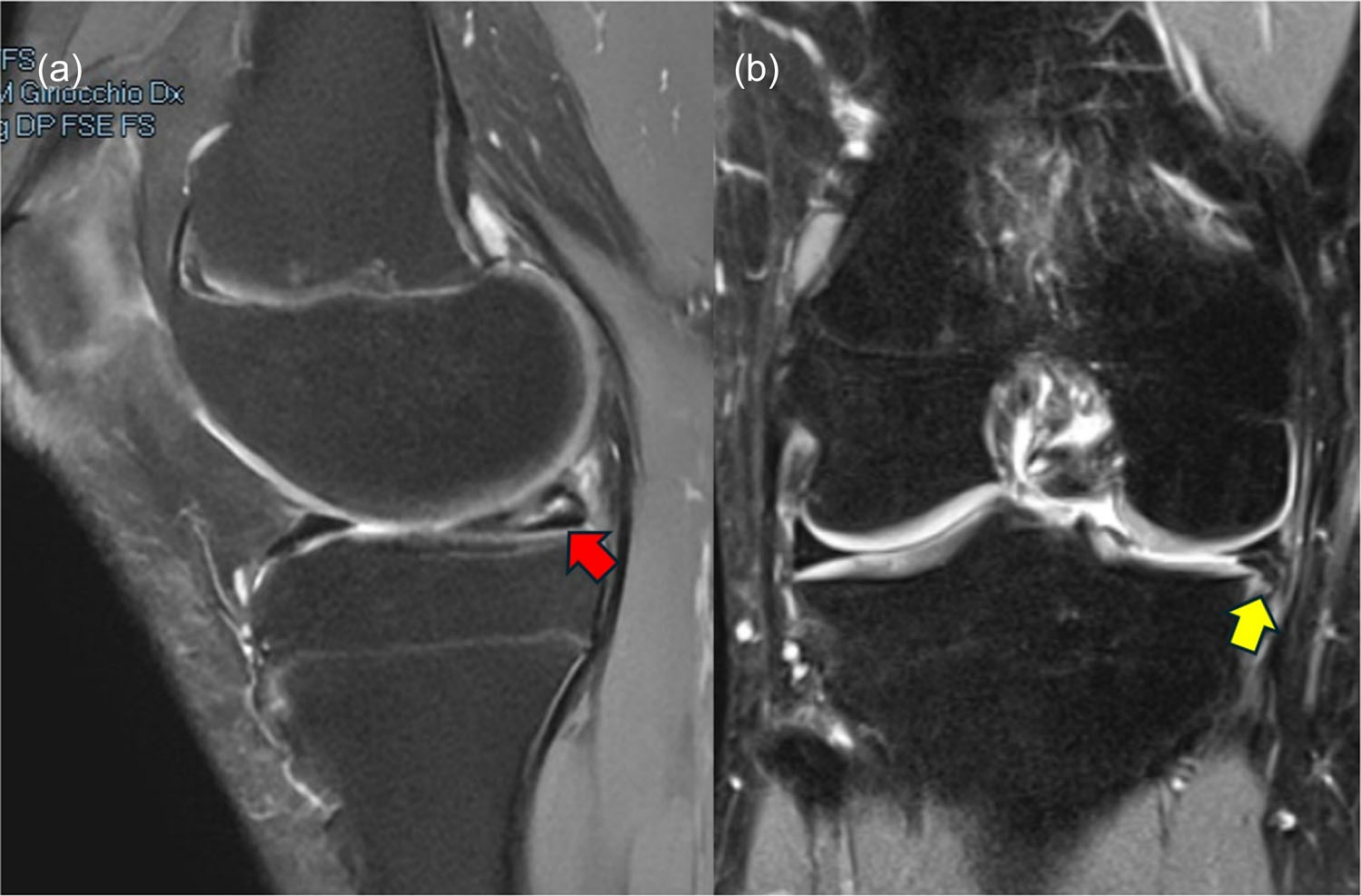
MRI features of degenerative meniscal lesions. (a) Horizontal cleavage tear. (b) Displaced flap tear.

MRI provides definitive visualization of horizontal cleavage tears typical of ageing (Figure 5). However, these lesions could be observed not only as isolated findings, but also as part of the broader structural changes associated with knee OA, with the presence of early cartilage degeneration and subtle alterations in joint mechanics even when radiographic OA appears mild or absent [16]. MRI therefore plays a central role in characterizing the extent and pattern of meniscal deterioration within the disease continuum. The ROAMES classification (Rapid Osteoarthritis MRI Eligibility Score) provides a structured MRI‐based framework to stratify OA phenotypes, helping to identify patients with more advanced structural involvement despite low Kellgren–Lawrence (KL) grades [44]. Within ROAMES, meniscal degeneration is graded from Grade 0 (normal), Grade 1 (increased intrameniscal signal without tear), Grade 2 (degenerative horizontal/complex tear without extrusion), to Grade 3 (degenerative tear with ≥3 mm extrusion), reflecting progressive loss of meniscal integrity and function. When interpreted with other MRI markers such as cartilage thinning, bone marrow lesions, osteophytes and effusion‐synovitis, this classification supports the identification of distinct OA phenotypes with different prognostic and therapeutic implications [45].

Standing long‐leg radiographs should also be obtained in order to evaluate limb alignment. Varus alignment increases medial compartment loading and accelerates degenerative change, whereas valgus deformity predisposes to lateral meniscal stress. Correct identification of malalignment, which is usually considered in the case of >4° deviation from the neutral mechanical axes, is crucial when considering mechanical offloading strategies or surgical options. This bilateral weight bearing radiograph is also useful to assess eventual side‐to‐side differences in terms of joint space narrowing and KL grading, in order to discern isolated meniscal lesions from low‐grade OA.

### Functional assessment

Beyond structural imaging, functional performance and ‘fitness’ assessment is helpful to quantify the patient's musculoskeletal reserve and guides rehabilitation. The 30‐Second Sit‐to‐Stand Test (30s‐STS) is a simple and reliable measure of lower‐limb strength and endurance [26]. Patients are instructed to stand up and sit down from a standard chair as many times as possible within 30 s, without using their arms [6] (Table 3). Scores below normative thresholds suggest quadriceps weakness and reduced physical capacity, both of which are associated with worse outcomes before and after meniscal injury or arthroscopic surgery [3, 50].

**Table 3:** Suggested normative values for Sit‐to‐Stand tests by age decade.

Age (years)	30‐s Sit‐to‐Stand (repetitions)	5‐rep Sit‐to‐Stand (seconds)
20–29	31–36 (typical ~33)	5.5–6.5 (typical ~6.0)
30–39	29–34 (typical ~31)	6–7.5 (typical ~6.5)
40–49	26–31 (typical ~28–29)	6–9 (typical ~7.5)
50–59	24–28 (typical ~26)	6–11 (typical ~7.7)
60–69	14–18 (screening: <12–14 below avg)	9–11 (age‐expected)
70–79	12–16 (screening: <10–12 below avg)	11–13 (age‐expected)
80–89	9–13 (screening: <7–9 below avg)	13–15 (age‐expected)

*Note*: Normative values for Sit‐to‐Stand (STS) tests by age decade. Reference values for 30‐s Sit‐to‐Stand (30s‐STS) and 5‐repetition Sit‐to‐Stand (5STS) tests.

An alternative to the 30s‐STS test is represented by the 5‐repetition sit‐to‐stand (5STS). During the test, the individual is instructed to stand up from a standard chair and sit down five times as quickly as possible without using the arms for support, while the total time to complete the task is recorded (Table 3). Longer completion times indicate reduced quadriceps strength and poorer neuromuscular control.

### Psychological assessment

In patients with degenerative meniscal lesions (‘meniscal ageing’), symptom burden often exceeds what structural imaging alone would predict. A targeted psychological assessment should therefore accompany clinical and imaging evaluation, as fear‐avoidance cognitions, anxiety/depression, catastrophizing and kinesiophobia are robustly associated with higher pain intensity and disability across musculoskeletal conditions. Waddell et al. demonstrated that fear‐avoidance beliefs independently relate to work loss and disability in back pain, introducing a biopsychosocial model for pain in degenerative conditions [56].

To investigate and document this psychological aspect, dedicated patient‐reported outcome measures have been created. The Pain Catastrophizing Scale (PCS; 13 items) is one of the most used and quantifies maladaptive pain cognitions, predicting worse outcomes after musculoskeletal injury. Typical interpretation treats higher total scores as clinically meaningful risk, guiding targeted education and cognitive strategies. The Tampa Scale of Kinesiophobia (TSK; 17 items) indexes fear‐avoidance; scores ≥37 are often used to denote high fear. Together, PCS and TSK help identify patients in whom central mechanisms and fear‐avoidance maintain pain despite largely age‐related meniscal change [57]. Recent clinical work in patients with meniscal injury shows that kinesiophobia correlates with knee pain intensity, self‐efficacy, functional status and balance; these data reinforce that psychological factors modulate symptom expression in meniscal pathology beyond the structural lesion itself. Thus, incorporating routine PCS/TSK screening into the assessment of degenerative meniscal pain is therefore warranted, particularly when MRI shows horizontal cleavage ‘ageing’ changes without clear mechanical block [51].

## PATIENT CATEGORIZATION ACCORDING TO COMPREHENSIVE ASSESSMENT

Not all degenerative meniscal lesions are clinically equivalent. Although they may appear similar on MRI, their symptom expression, prognosis and therapeutic relevance can vary widely depending on the biological context and mechanical environment of the knee [16, 23]. Thus, the same meniscal lesion in two different patients may behave either as a harmless sign of physiological meniscal ageing or as part of a pathological degenerative cascade [15, 43]. In knee OA, this concept of phenotyping, which aims to classify patients into subgroups based on clinical characteristics, metabolic status, synovial activity and anatomical damage, has become central to understanding mechanisms and tailoring treatment [10, 11]. Six phenotypes have been described for knee OA, including (1) chronic pain phenotype with central sensitization, (2) inflammatory phenotype, (3) metabolic syndrome phenotype, (4) bone and cartilage metabolism phenotype, (5) mechanical (malalignment) phenotype and (6) minimal joint disease phenotype [10]. Given that the meniscus plays a key role in the initiation of osteoarthritic change, a similar phenotypic framework could be applied to degenerative/ageing‐related meniscal lesions; clustering meniscal ageing and degenerative lesions into clinical patterns could help the clinician understand prognosis and guide the therapeutic tone. Based on clinical examination, metabolic status, alignment, functional testing and MRI features, four broad ageing‐related meniscal categories could be suggested:

1. Physiological or Asymptomatic Meniscal Ageing (Physiological Pattern): In many adults over the age of 40, horizontal intrameniscal signal changes or small degenerative cleavages are incidental findings, particularly when MRI is performed for non‐meniscal symptoms. These individuals demonstrate preserved alignment, no meniscal extrusion, minimal synovitis and normal functional performance. Here, the meniscal lesion represents ageing rather than disease, and reassurance, education and maintenance of lower‐limb strength are generally sufficient.
2. Metabolic/Inflammatory Susceptible Meniscal Degeneration (Metabolic Pattern): In patients with high BMI or obesity, dyslipidemia, insulin resistance, vitamin D deficiency or low‐grade systemic inflammation, meniscal degeneration may become symptomatic even in the absence of significant mechanical overload. The internal tissue vulnerability and altered nociceptive sensitivity create a joint environment in which degenerative meniscal lesions are amplifiers of pain rather than primary mechanical problems. In this phenotype, metabolic optimization and systemic risk modification are fundamental components of care.
3. Mechanical Overload–Driven Meniscal Degeneration (Mechanical Pattern): In individuals with varus or valgus malalignment, elevated body weight, small medial femoral condyles or high cumulative loading from sport or occupational kneeling, the ageing meniscus becomes overwhelmed by excess compartmental stress. These patients frequently demonstrate meniscal extrusion, subchondral bone marrow lesions and early compartmental cartilage thinning. The biomechanical driver dominates and management strategies focus on load redistribution and neuromuscular strengthening, occasionally advancing to corrective osteotomy when malalignment is structurally significant.
4. Meniscal Degeneration within Established Osteoarthritis (Advanced Pattern): In more advanced cases (often KL ≥ 2), the degenerative meniscal tear reflects the joint‐level degenerative process, rather than an isolated target for intervention. Symptoms derive from the interplay of cartilage loss, synovitis, bone marrow lesion activity and altered gait loading. In this context, the meniscus serves as a marker of disease state and treatment aligns with comprehensive OA management rather than focal meniscal repair or resection.

**Figure 6:**
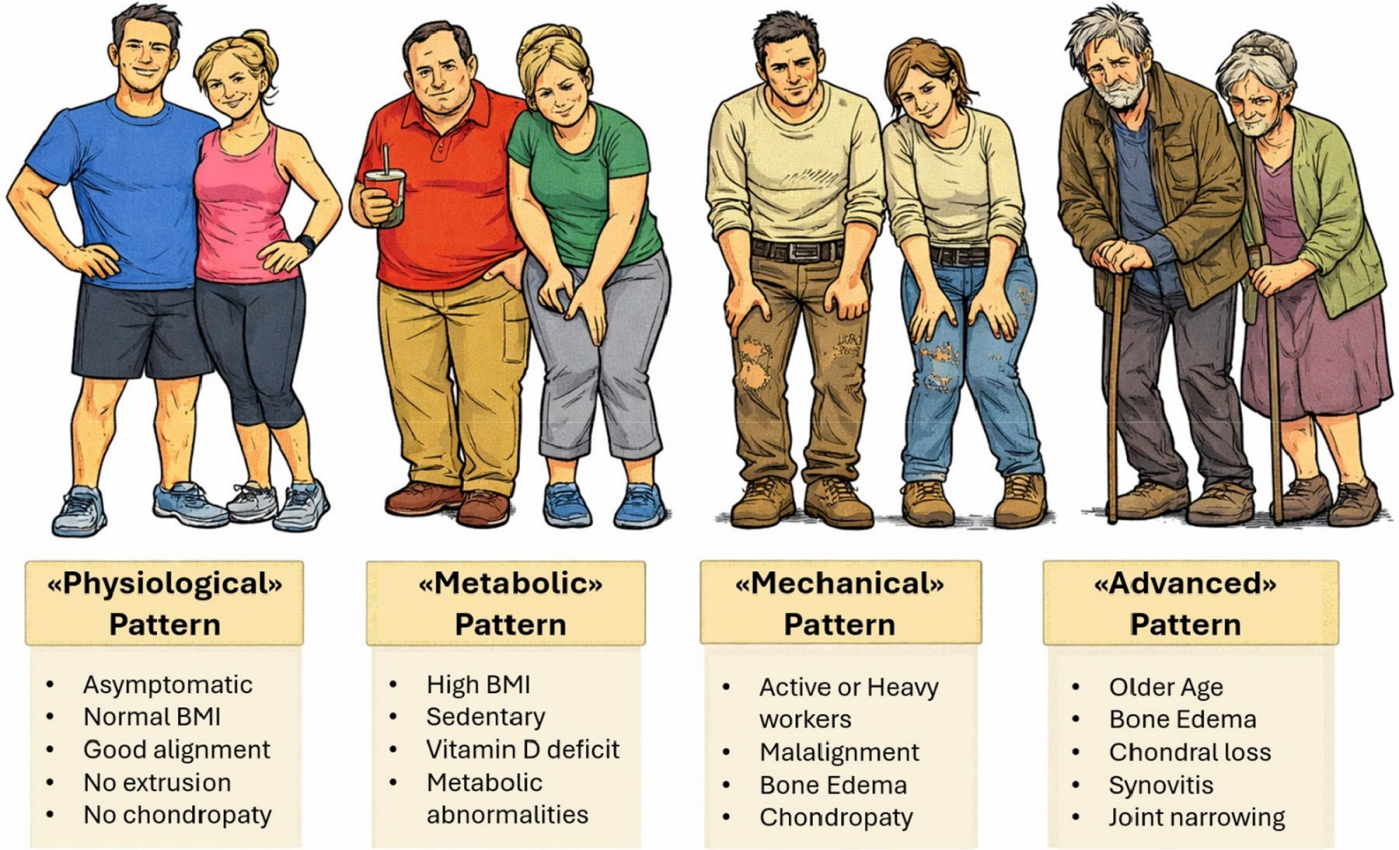
Based on clinical findings, metabolic profile, limb alignment, functional performance and MRI characteristics, four broad phenotypic patterns of ageing‐related meniscal degeneration can be identified: (1) Physiological pattern of meniscal ageing, (2) Metabolic/Inflammatory susceptible pattern, (3) Mechanical overload degeneration pattern and (4) Advanced/Osteoarthritic pattern. MRI, magnetic resonance imaging.

This proposal for framing reinforces that meniscal ageing is not inherently pathological, but may become so when combined with metabolic sensitivity, mechanical overload or global joint degeneration (Figure 6). Identifying the correct condition shapes expectations, guides conversations with the patient and informs subsequent decisions on conservative, biologic or surgical pathways—topics that will be addressed in the following section.

## INTEGRATED CONSERVATIVE MANAGEMENT

Many horizontal cleavage tears, particularly those detected incidentally on MRI, represent the structural ‘wrinkles’ of meniscal ageing. In such cases of ‘physiological pattern’, no surgical treatment is warranted. Patients should be reassured regarding the benign nature of the finding and counselled on optimizing modifiable factors—maintaining a healthy body weight, ensuring Vitamin D sufficiency and practicing regular low‐impact exercise such as cycling, walking or swimming.

Follow‐up should be clinical rather than radiographic unless new symptoms develop. This phenotype exemplifies the principle of ‘treat the patient, not the picture’. Also in patients with the ‘Metabolic pattern’, with mild symptoms and biochemical abnormalities (hyperlipidemia, vitamin D deficiency, metabolic syndrome), surgical intervention is rarely indicated. The emphasis lies on lifestyle modification—dietary counselling to reduce saturated fats and improve omega‐3 intake, weight loss and regular physical activity. Pharmacological optimization, including statin therapy for dyslipidemia or Vitamin D supplementation, can contribute to joint homoeostasis. When pain persists despite metabolic correction, symptomatic treatments such as hyaluronic acid (HA) or PRP injections should be considered before contemplating surgery.

Following, a multimodal conservative management of the ageing meniscus which combines mechanical, metabolic and biological strategies is described in detail. Conservative care should be trialed for at least 3 months before considering surgery (Table 4) [2, 42].

**Table 4:** Integrated management strategies for degenerative (‘ageing’) meniscal lesions.

Management domain	Indications	Practical intervention	Description
Reassurance & Observation	Physiological pattern; incidental MRI findings; minimal or no symptoms	Patient education, reassurance, clinical follow‐up only	Horizontal cleavage tears often represent age‐related structural changes
Lifestyle Optimization	Physiological or metabolic pattern	Weight control, low‐impact aerobic activity (walking, cycling, swimming)	Reduces joint load and supports joint homoeostasis; first‐line intervention
Exercise Therapy & Rehabilitation	All symptomatic degenerative meniscal lesions	Supervised physiotherapy, quadriceps, hip abductors, balance training	Improves neuromuscular control and reduces pain
Analgesia	Mild–moderate pain	Intermittent NSAIDs (e.g., ibuprofen 400–600 mg up to TID for 5–7 days)	Symptomatic relief; adjunct to exercise therapy
Diet & Metabolic Health	Metabolic pattern; overweight patients	Anti‐inflammatory diet; weight reduction (5–10% body mass)	5–10% weight loss reduces knee joint load by 20%–30%
Collagen Supplementation	Degenerative meniscal lesions ± early OA	Collagen peptides 10 g/day for 2–3 months	RCT evidence of pain and QoL improvement; adjunctive role
Vitamin D Supplementation	Serum 25(OH)D < 30 ng/mL	Vitamin D_3_ 800–2000 IU/day or 50,000 IU monthly; repletion if deficient	Associated with reduced MRI progression of cartilage and meniscal damage
Cholesterol Management	Hyperlipidemia; metabolic pattern	Lifestyle modification ± statins	Elevated lipids predict bone marrow lesions and OA progression
Mechanical Offloading	Medial meniscal degeneration or extrusion	Lateral wedge insoles	Reduces medial compartment load and meniscal extrusion
Shockwave Therapy (ESWT)	Persistent pain after first‐line conservative care	Focused ESWT (0.25 mJ/mm^2^, 2000 impulses, weekly ×3)	RCTs show reduced pain and improved MRI biomarkers
Psychological Interventions	High PCS/TSK scores	Pain education, cognitive‐behavioural strategies, graded exposure	Addresses fear‐avoidance and catastrophizing; improves pain and function
Intra‐articular Injections—Corticosteroids	Inflammatory or metabolic pattern; OA flares	1–2 intra‐articular triamcinolone acetonide 40 mg	Short‐term pain relief; low cost and widely available
Intra‐articular Injections—Hyaluronic Acid	Mechanical pattern	2–3 intra‐articular injections (2 mL, 1–1.5%), every 2–4 weeks	Improves lubrication; pain relief up to 6 months in selected patients
Intra‐articular Injections—PRP	Persistent symptoms; younger or active patients	1–3 intra‐articular injections (4–6 mL), 2 weeks apart	Meta‐analyses show superiority over HA, especially leucocyte‐poor PRP
Perimeniscal Injections	Meniscal extrusion; localized pain	1 perimeniscal corticosteroid injection	High surgery‐free survival; effective when combined with physiotherapy
Arthroscopic Partial Meniscectomy	Mechanical symptoms (locking/catching) after ≥3 months conservative care	Selective removal of unstable fragments	Reserved indication; risk of accelerated OA; preserve meniscal rim
Meniscal Repair	Young, active patients; good cartilage and alignment	Arthroscopic repair (e.g., root or radial tears)	Reoperation or persistent pain in 20%–30%; prolonged rehab required
Joint‐Preserving Surgery	Mechanical pattern; malalignment >4°	HTO or DFO ± meniscal root repair	Restores biomechanics; delays OA progression and arthroplasty
Joint‐Replacing Surgery	Advanced pattern; KL Grade 4 OA	UKA or TKA	Meniscal lesion is epiphenomenon; treatment targets global joint failure

*Note*: Summary of conservative and surgical treatment strategies for degenerative meniscal lesions, according to clinical pattern and symptom profile, ranging from observation and lifestyle optimization to joint‐preserving procedures and ultimately joint replacement surgery in advanced disease.

Abbreviations: DFO, distal femoral osteotomy; ESWT, extracorporeal shockwave therapy; HA, hyaluronic acid; HTO, high tibial osteotomy; KL, Kellgren–Lawrence; MRI, magnetic resonance imaging; NSAIDs, nonsteroidal anti‐inflammatory drugs; OA, osteoarthritis; PRP, platelet‐rich plasma; PCS/TSK, Pain Catastrophizing Scale/Tampa Scale of Kinesiophobia; QoL, quality of life; RCT, randomized controlled trial; TKA, total knee arthroplasty; UKA, unicompartmental knee arthroplasty.

### Physical activity and rehabilitation

Exercise therapy represents the primary treatment for degenerative meniscal lesions. It restores neuromuscular control, enhances joint stability and reduces load on degenerated tissue. Quadriceps and hip abductor strengthening are particularly beneficial, as weakness in these groups correlates with knee pain and OA progression [43]. A pragmatic regimen includes supervised physiotherapy sessions 2–3 times weekly for 8–12 weeks, focusing on quadriceps eccentric and isometric strengthening (leg press or chair, three sets of 10–12 reps), gluteal and hamstring activation, balance and proprioceptive drills, gradual return to aerobic activity (cycling, elliptical training, swimming). After 12 weeks, maintenance exercises should be continued at home thrice weekly [28]. Patients with mild pain may use non‐steroidal anti‐inflammatory drugs (NSAIDs) intermittently (e.g. ibuprofen 400–600 mg up to three times daily for 5–7 days as needed).

### Diet and metabolic health

Nutritional optimization contributes to both symptom control and structural preservation. Diets low in saturated fat and high in omega‐3 fatty acids, fruits and vegetables are associated with lower systemic inflammation and improved cartilage metabolism. Weight reduction is crucial: even a 5%–10% body mass decrease lowers knee joint load by 20%–30%. Patients should receive counselling on caloric balance and physical activity adherence.

### Collagen supplements

Collagen peptide supplementation is an emerging adjunct. In a randomized, double‐blind trial, Genç et al. [21] found that 8 weeks of combined Type I/III and Type II collagen hydrolysate (10 g/day orally) improved pain and quality of life in patients with degenerative meniscal pathology. Collagen peptides may enhance cartilage matrix metabolism through increased hydroxyproline availability and anti‐inflammatory cytokine modulation. Clinically, a daily 10 g oral dose for 2–3 months is reasonable, particularly in older adults with concomitant OA or sarcopenia.

### Vitamin D supplementation

Vitamin D regulates chondrocyte metabolism and inflammatory signalling. Joseph et al. [27] reported that higher vitamin D intake correlated with less progression of cartilage and meniscal MRI abnormalities. For adults, maintaining serum 25(OH)D levels above 30 ng/mL is advisable. Typical supplementation involves cholecalciferol (Vitamin D_3_) 800–2000 IU daily, or 50.000 IU monthly, with calcium intake of 1000–1200 mg/day from dietary sources. In deficient patients (<20 ng/mL), a repletion regimen of 50.000 IU weekly for 6–8 weeks may be used before maintenance.

### Cholesterol management

Hyperlipidemia is increasingly recognized as a modifiable risk factor for joint degeneration. Davies‐Tuck et al. [9] demonstrated that higher serum cholesterol and triglycerides predicted incident bone marrow lesions—markers of early OA. Clinicians should address lipid control in all middle‐aged patients with degenerative meniscal changes. Lifestyle interventions (diet, exercise) are first‐line; pharmacological treatment follows standard cardiovascular guidelines, typically atorvastatin 10–20 mg/day or equivalent where indicated. Optimizing lipid profiles may indirectly support meniscal and subchondral health.

### Foot insoles and mechanical offloading

For patients with medial meniscal degeneration or extrusion, laterally wedged insoles (LWI) help redistribute tibiofemoral loads. Finite‐element modelling demonstrates that 5°–10° lateral wedges reduce medial compartment stress [30]. Ultrasonography confirms that LWI significantly decreases medial meniscus extrusion during stance. Clinically, a 5° lateral wedge insole inserted into stable footwear is recommended. It should be worn during weight‐bearing activities for 6–8 h per day, reassessed after 6 weeks. Patients often report reduced medial joint pain and improved tolerance for walking [30].

### Shockwave therapy

Extracorporeal shockwave therapy (ESWT) is a non‐invasive adjunct promoting neovascularization and anti‐inflammatory responses. Hashimoto et al. [25] conducted a randomized clinical trial showing that focused ESWT (0.25 mJ/mm^2^, 2000 impulses, weekly × 3 sessions) reduced meniscal T2 relaxation times and knee pain at 12 months. Thus, ESWT can be offered to patients with persistent degenerative meniscal pain after failed conservative measures. Treatment is generally well tolerated and can be combined with exercise therapy.

### Psychological interventions

When elevated PCS and TSK values are identified, cognitive‐behavioural pain education, graded exposure to feared tasks and self‐efficacy strategies could be integrated alongside exercise therapy and load management. Evidence syntheses of the fear‐avoidance model show that reducing catastrophizing and fear may improve pain and function [56, 57], since beliefs are often linked to disabilities and symptom persistence could be triggered by psychological processes. The PCS and the TSK could be used to monitor and target cognitive reframing within the rehabilitation plan.

### Intra‐articular (IA) injection

IA injections provide symptom relief and facilitate rehabilitation in patients with persistent pain. Different products are available to treat the ageing meniscus and degenerative lesions.

#### Corticosteroids

It is considered first‐line treatment, especially in patients with metabolic and inflammatory pattern because of its availability, safety and limited cost. Short‐term corticosteroid injections (e.g., triamcinolone acetonide 40 mg in 1 mL) may be appropriate for inflammatory flares or concomitant OA. A common protocol consists in two consecutive injections separated by 2–4 weeks, usually associated with joint aspiration in the case of abundant swelling [34].

#### Hyaluronic acid (HA)

Viscosupplementation restores synovial lubrication in patients with mechanical pattern, but may exert anti‐inflammatory effects as well. Randomized trials report modest but clinically meaningful benefits in selected patients. Different compositions and regimens are available, with the most common consisting of 2–3 IA injections of 2 mL 1%–1.5% HA, every 2‐4 weeks. Pain relief typically begins within 2–4 weeks and may last up to 6 months [34].

#### Platelet‐rich plasma (PRP)

Autologous PRP provides growth factors that modulate joint inflammation and promote matrix synthesis. Meta‐analyses suggest superior pain and function outcomes compared to HA, particularly with leucocyte‐poor PRP preparations [19, 34]. No gold‐standard protocol exists: usually 1–3 injections of 4–6 mL each, spaced 2 weeks apart, are performed under sterile conditions. Post‐injection rest for 24–48 h is advised before resuming physiotherapy.

#### Perimeniscal injections

Targeted ultrasound‐guided perimeniscal injections have emerged as an effective minimally invasive treatment. Di Sante et al. [12] demonstrated significant pain reduction after perimeniscal corticosteroids in patients with medial extrusion. Duprat et al. [14] reported 95% surgery‐free survival at 24 months following a single ultrasound‐guided injection of triamcinolone acetonide 40 mg mixed with 1–2 mL of 1% lidocaine, with significant improvement in rest and walking pain. Mabrouk et al. [31] achieved similar results combining perimeniscal injection with structured physiotherapy, consisting of clinical success in 83% of 671 patients at 5 years. A practical regimen involves one perimeniscal corticosteroid injection, followed by physiotherapy starting within 1 week, while a repeat injection may be considered after 6–9 months if symptoms recur.

## SURGICAL MANAGEMENT

A minority of patients with meniscal pathology and degenerative tears present with acute mechanical symptoms such as locking or catching, typically due to a displaced flap or unstable fragment trapped between the medial tibial plateau and the medial collateral ligament (Figure 5b). MRI commonly reveals a flap or bucket‐handle morphology superimposed on degenerative changes. These lesions act like ‘a stone in your shoe′ but may progressively disappear over time by auto‐disintegration. If this does not happen, arthroscopic partial meniscectomy remains appropriate, only after 3‐month trial of conservative treatment.

### Arthroscopic partial meniscectomy

The procedure should be limited to removal of unstable fragments while preserving as much peripheral rim as possible to maintain hoop tension. It can be compared to the removal of the small ‘stone in the shoe’ trying not to damage the foot and the shoe. Excessive resection should be avoided, particularly in the posterior horn, where loss of tissue significantly compromises hoop stress transmission [20, 23]. Postoperative rehabilitation begins immediately, with full weight‐bearing as tolerated and progressive strengthening.

Outcomes are generally favourable in properly selected cases, but clinicians must counsel patients about potential complications because of loss of meniscus function—either due to the lesion itself and the surgical removal—which include accelerated cartilage wear, subchondral osteonecrosis and increased long‐term OA risk [53]. Radiographic progression of OA after the occurrence of degenerative meniscus lesions necessitating surgical intervention has been reported in up to 40% of patients at 5 years [18].

### Arthroscopic meniscal repair

In the case of symptomatic horizontal, radial or longitudinal tears with otherwise healthy cartilage and normal alignment in young and active patients, meniscal repair may be considered if vascularity and tissue quality permit.

Meniscal repair techniques include all‐inside, inside‐out and outside‐in approaches, each selected according to tear location and morphology.

The all‐inside technique utilizes preloaded suture devices deployed entirely within the joint. It is particularly suited for posterior horn tears, where the design of the devices facilitates access and allows a more straightforward and reproducible repair. In many cases, multiple sutures are often required to achieve adequate stability [54].

The inside‐out technique remains a widely used alternative for the treatment of posterior horn lesions, offering reliable fixation. However, it requires a meticulous surgical approach due to the risk of neurovascular injury, particularly involving the saphenous nerve on the medial side and the common peroneal nerve on the lateral side [52].

The outside‐in technique is especially indicated for anterior horn and mid‐body tears. In this approach, two 18‐gauge spinal needles are percutaneously introduced at the meniscocapsular junction. Through one needle, a free absorbable monofilament suture is advanced into the joint, while through the second needle a suture loop is introduced. Under arthroscopic visualization, the free suture is passed into the loop and retrieved by pulling the loop out, allowing controlled shuttling of the suture through the meniscus. Sutures are then tied over the capsule through small skin incisions [39].

Regardless of the technique, clinicians should discuss the risk of reoperation or persistent pain, reported in up to 20%–30% of cases [2, 32] and prolonged recovery which could require 3–4 months of structured rehabilitation focusing on gradual range‐of‐motion and strengthening.

### Joint‐preserving surgical procedures

The group of patients with the ‘mechanical pattern’ includes those with symptomatic medial or lateral compartment overload, often characterized by varus or valgus malalignment >4°, meniscal extrusion, high body weight or small medial compartments and cartilage wear confined to one compartment. In these cases, surgery should aim to restore joint biomechanics rather than simply remove degenerated tissue. High tibial osteotomy (HTO) for varus deformity or distal femoral osteotomy (DFO) for valgus alignment can effectively redistribute load away from the compromised compartment. Multiple studies have demonstrated that alignment correction reduces pain, delays osteoarthritic progression and postpones knee replacement [8]. When a posterior root tear coexists with varus malalignment, combining root repair with HTO could theoretically offer a superior joint preservation compared with isolated procedures [1]. Root repair is typically performed using a transtibial pull‐out technique with sutures passed through the anatomic footprint or with all‐inside repair. Postoperative rehabilitation involves non‐weight‐bearing for 4–6 weeks, followed by gradual return to full weight‐bearing by 8–10 weeks and 3–4 months of physiotherapy before resuming sports.

### Joint‐replacing surgical procedures

Patients with the ‘advanced pattern’ include those where degenerative meniscal lesions coexist with advanced radiographic OA, defined as KL Grade 4 or extensive full‐thickness cartilage loss. In these patients joint preservation becomes unrealistic, since the meniscal lesions are merely an epiphenomenon of global articular collapse.

Surgical management should therefore target the joint as a whole: in patients with isolated unicompartmental disease (e.g., medial OA, varus alignment <10°, intact cruciate ligaments), unicompartmental knee arthroplasty (UKA) provides excellent outcomes and faster recovery compared with total replacement. For those with bicompartmental or tricompartmental OA, or significant deformity, total knee arthroplasty (TKA) remains the gold standard. Both procedures effectively address pain and restore function, especially in bone‐on‐bone scenarios and in the case of deformities [22]. However, the UKA may preserve proprioception and allow quicker return to activity. The presence of an ‘ageing meniscus’ in this context does not require separate intervention.

## CONCLUSION

The ageing meniscus embodies the continuum of joint senescence. Horizontal degenerative lesions, which are the typical manifestation of this process, should be viewed as ‘wrinkles’ in the knee joint rather than injuries. They are frequently asymptomatic and often discovered incidentally.

The modern orthopaedic approach must prioritize preservation, counselling and patient education over surgical intervention. Most patients benefit from a comprehensive conservative strategy combining physical activity, metabolic optimization and injective therapies. Surgery should be reserved for selected cases with persistent mechanical symptoms or structural instability.

In the context of an ageing population, recognizing the meniscal lesion as an expression of biological rather than pathological ageing marks an essential step toward rational, patient‐centred knee care.

## AUTHOR CONTRIBUTIONS

Alberto Grassi and Stefano Zaffagnini conceived and designed the study. Alberto Grassi and Claudio Rossi drafted the manuscript. Stefano Zaffagnini and Peter Verdonk supervised the project and critically revised the manuscript. All authors interpreted the data, contributed to manuscript revision and approved the final version of the manuscript.

## CONFLICT OF INTEREST STATEMENT

Alberto Grassi—Smith & Nephew: Not paid consultant. Stefano Zaffagnini—DePuy, a Johnson & Johnson Company: Paid presenter or speaker; paid consultant; European Society of Sports Traumatology, Knee Surgery and Arthroscopy (ESSKA): Board or committee member International Society of Arthroscopy, Knee Surgery and Orthopaedic Sports Medicine (ISAKOS): Board or committee member; *Journal of Experimental Orthopaedics* (*JEO*): Editorial or governing board. Smith & Nephew: Paid presenter or speaker; paid consultant. Verdonk Peter—Royalties received from Adler, CONMED Linvatec. Speaker for Active Implants, CONMED Linvatec Paid Consultant for Active Implants, CONMED Linvatec. Support received from Cartiheal. Editorial or Governing board of *KSSTA* journal. Board of Directors member for International Society of Arthroscopy, Knee Surgery and Orthopaedic Sports Medicine, ESSKA, ICRS. The remaining author declares no conflict of interest.

## ETHICS STATEMENT

The authors have nothing to report.

## Data Availability

The authors have nothing to report.
